# Real-Time Prediction of Sepsis in Critical Trauma Patients: Machine Learning–Based Modeling Study

**DOI:** 10.2196/42452

**Published:** 2023-03-31

**Authors:** Jiang Li, Fengchan Xi, Wenkui Yu, Chuanrui Sun, Xiling Wang

**Affiliations:** 1 School of Public Health and Key Laboratory of Public Health Safety Fudan University Shanghai China; 2 Research Institute of General Surgery Affiliated Jinling Hospital Medical School of Nanjing University Nanjing China; 3 Department of Intensive Care Unit Affiliated Drum Tower Hospital Medical School of Nanjing University Nanjing China

**Keywords:** sepsis, trauma, intensive care unit, machine learning, real-time prediction

## Abstract

**Background:**

Sepsis is a leading cause of death in patients with trauma, and the risk of mortality increases significantly for each hour of delay in treatment. A hypermetabolic baseline and explosive inflammatory immune response mask clinical signs and symptoms of sepsis in trauma patients, making early diagnosis of sepsis more challenging. Machine learning–based predictive modeling has shown great promise in evaluating and predicting sepsis risk in the general intensive care unit (ICU) setting, but there has been no sepsis prediction model specifically developed for trauma patients so far.

**Objective:**

To develop a machine learning model to predict the risk of sepsis at an hourly scale among ICU-admitted trauma patients.

**Methods:**

We extracted data from adult trauma patients admitted to the ICU at Beth Israel Deaconess Medical Center between 2008 and 2019. A total of 42 raw variables were collected, including demographics, vital signs, arterial blood gas, and laboratory tests. We further derived a total of 485 features, including measurement pattern features, scoring features, and time-series variables, from the raw variables by feature engineering. The data set was randomly split into 70% for model development with stratified 5-fold cross-validation, 15% for calibration, and 15% for testing. An Extreme Gradient Boosting (XGBoost) model was developed to predict the hourly risk of sepsis at prediction windows of 4, 6, 8, 12, and 24 hours. We evaluated model performance for discrimination and calibration both at time-step and outcome levels. Clinical applicability of the model was evaluated with varying levels of precision, and the potential clinical net benefit was assessed with decision curve analysis (DCA). A Shapley additive explanation algorithm was applied to show the effect of features on the prediction model. In addition, we trained an L2-regularized logistic regression model to compare its performance with XGBoost.

**Results:**

We included 4603 trauma patients in the study, 1196 (26%) of whom developed sepsis. The XGBoost model achieved an area under the receiver operating characteristics curve (AUROC) ranging from 0.83 to 0.88 at the 4-to-24-hour prediction window in the test set. With a ratio of 9 false alerts for every true alert, it predicted 73% (386/529) of sepsis-positive timesteps and 91% (163/179) of sepsis events in the subsequent 6 hours. The DCA showed our model had a positive net benefit in the threshold probability range of 0 to 0.6. In comparison, the logistic regression model achieved lower performance, with AUROC ranging from 0.76 to 0.84 at the 4-to-24-hour prediction window.

**Conclusions:**

The machine learning–based model had good discrimination and calibration performance for sepsis prediction in critical trauma patients. Using the model in clinical practice might help to identify patients at risk of sepsis in a time window that enables personalized intervention and early treatment.

## Introduction

Sepsis is a life-threatening type of organ dysfunction caused by a dysregulated host response to an infection [1]. It is a major contributor to the global burden of disease, with morbidity and mortality rates having failed to decrease substantially during the past decade, especially in the trauma population [2,3]. According to the international consensus guidelines for sepsis, fluid resuscitation should commence within the first 3 hours of sepsis, and antimicrobial treatment should commence within 1 hour of sepsis [4-6]. The mortality rate of sepsis increases significantly with each hour of delayed administration of antibiotics [5,6]. However, early recognition of sepsis can be challenging due to the complexity of the sepsis response and the heterogeneity of the population with sepsis [7,8]. Furthermore, delays in communication among health care providers may exacerbate sepsis-management delays [9]. Therefore, closely evaluating and predicting the risk of sepsis before onset at an individual level may provide insights for clinicians to implement timely personalized medicine to improve prognoses.

The traditional tools to predict sepsis are often based on generalized linear models. The Epic Sepsis Model (ESM), a penalized logistic regression model, is one of the most widely implemented early warning systems for sepsis, especially in the United States. However, Wong et al [10] recently found that the ESM had poor discrimination performance, with an area under the receiver operating characteristics curve (AUROC) of 0.76 to predict sepsis 4 hours in advance; it also failed to detect sepsis before its onset in 67% of patients. Machine learning–based predictive modeling is increasingly popular and is being applied in clinical research and practice due to the availability of large digitized medical data sets and computing power [11,12]. The advantage of machine learning algorithms lies in their capability to extract the most important information from complex data and capture nonlinear relations between features. Machine learning models, including gradient boosting trees, random forests, and neural networks, have been developed for real-time prediction of sepsis or sepsis shock in a general intensive care unit (ICU) setting [13,14].

However, to our knowledge, there is no such real-time prediction model aimed specifically at the trauma population. Unlike general patients, most trauma patients are relatively young, are predominantly male, and have few underlying medical conditions [2,15]. The weight of these factors in the prediction models for trauma patients might differ from the weights in models developed for other critical patients. Furthermore, a hypermetabolic baseline and explosive inflammatory immune response mask clinical signs and symptoms of sepsis in trauma patients, making it more difficult to diagnose sepsis in the early stages [16,17]. Therefore, the development of a real-time prediction model for sepsis in the trauma population would be clinically valuable and could help clinicians to identify patients at high risk of developing sepsis, leading to improved medical care [13]. In this study, we aimed to develop a machine learning model using Extreme Gradient Boosting (XGBoost) and a publicly available database to predict the risk of sepsis at an hourly scale in trauma patients admitted to an ICU.

## Methods

### Data Source

Data were obtained from a publicly available database, the Medical Information Mart for Intensive Care IV (MIMIC IV; version 1.0), which continuously collected medical records from the ICU at Beth Israel Deaconess Medical Center (Boston, MA) between 2008 and 2019 [18].

### Patient Selection and Variable Extraction

All patients aged ≥18 years in the database who had a first-discharge diagnosis of trauma according to the ninth or tenth revisions of the International Classification of Diseases (ICD) codes (ICD-9: 800-848, 850-854, 860-887, 890-897, 900-904, 910-929, or 950-957; ICD-10: S00-S99) were included. In the case of multiple ICU admissions, we used only data from the first episode of ICU admission to avoid repeated measures of sepsis. Patients who developed sepsis before ICU admission were excluded. Medical records after the occurrence of sepsis were not used in the model development due to considerations of the clinical applicability of the model.

A total of 42 raw variables were chosen based on the previous literature and their clinical relevance. They were extracted based on an SQL search with Navicat Premium (version 15.0.21; PremiumSoft CyberTech Ltd) [13]. These features represented a mix of static and dynamic information. A full set of the variables is listed in Multimedia Appendix 1.

### Ethical Approval

This database was approved by the Beth Israel Deaconess Medical Center (45682859) [19]. The need for informed consent was waived because of the completely anonymous nature of the data and the retrospective nature of the study. We completed the relevant courses to access the database and obtained a certificate (45682859).

### Outcomes

Sepsis was defined as the presence of both suspected infection and organ dysfunction according to the recent sepsis-3 criteria [1,20]. The onset time of sepsis was defined as the earliest time of suspected infection and organ dysfunction, manifested as an acute increase in the Sequential Organ Failure Assessment (SOFA) score of at least 2 [21,22]. More details on the definition of the onset time of sepsis are provided in Multimedia Appendix 2.

### Data Preprocessing

**Figure 1:**
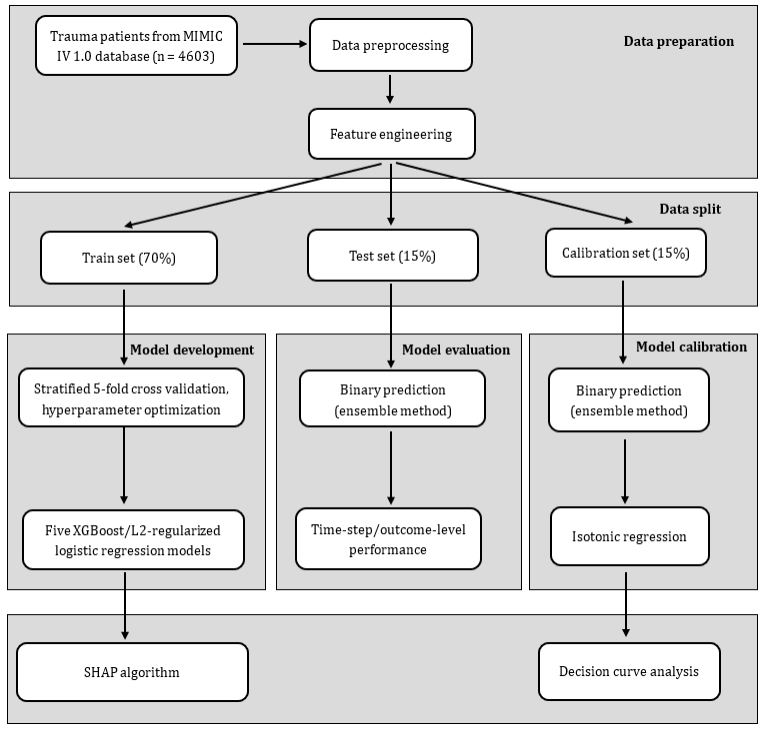
Flowchart of model development. MIMIC IV: Medical Information Mart for Intensive Care IV; SHAP: Shapley additive explanation.

To optimize the data for the model input, static variables were repeated at each 1-hour time grid. Dynamic variables measured more than once per hour were aggregated into 1-hour time steps by calculating hourly medians. We adopted the last-occurrence-carry-forward strategy to impute missing values for each variable. Population means were used for imputing the remaining missing values occurring before the first measurement [23]. A schematic workflow of the study is shown in Figure 1.

### Feature Engineering

A total of 485 features were derived from the raw variables, classified into three subtypes: (1) 37 measurement pattern features, (2) 7 scoring features, and (3) 441 time-series variables. Details of the feature engineering are described in Multimedia Appendix 3. Finally, a total of 527 features were used for model development.

### Model Development

The data set was randomly split into three sub–data sets: 70% for model training with stratified 5-fold cross-validation, 15% for calibration, and 15% for testing. Records for each patient, rather than individual time steps, were assigned to the same training, validation, calibration, and test sets to avoid label leaking. As just over 2% (529/26,140) of individual time steps presented to the model were labeled as sepsis, we remedied this imbalance in the data set by tuning parameters to change the weight between the positive and negative classes during the training process.

We used XGBoost, a gradient boosting algorithm well-known for obtaining winning solutions in various data competitions [24], to predict the risk of sepsis onset among trauma patients in the following prediction windows: 4, 6, 8, 12, and 24 hours; the temporal resolution was 1 hour. The choice of time windows was in accordance with previous literature predicting the risk of sepsis in general ICU patients [13,25] and takes into account the time needed before making interventions in clinical sepsis management, as well as prediction accuracy [26]. To reduce the risk of model overfitting, 5-fold cross-validation was used to produce 5 XGBoost models on the training set. Bayesian optimization was used to select the optimal hyperparameter combinations by maximizing AUROC in the validation set [27]. The ensemble method was used to provide robust estimation by averaging prediction probabilities from the above 5 models [28].

In addition, we trained an L2-regularized logistic regression model to compare its performance with XGBoost. Continuous features were standardized to improve the speed of model convergence before fitting. The grid search algorithm was applied to select the optimal strength of regularization. The ensemble approach was also adopted for the final prediction.

### Model Evaluation and Model Calibration

We evaluated model discrimination performance on the test set at both the time-step level and outcome level. At the time-step level, we calculated the AUROC and the area under the precision-recall curve (AUPRC) with prediction windows of 4, 6, 8, 12, and 24 hours for XGBoost and logistic regression. Sensitivity and specificity were calculated for prediction window/precision pairs (at 5%, 8%, and 10%) for XGBoost. At the outcome level, we computed sensitivity at different levels of precision. Unlike time-step–level sensitivity, outcome-level sensitivity corresponded to the percentage of all sepsis episodes that had at least one correct prediction within a specific time window before sepsis onset. Model calibration was evaluated with the average calibration error (ACE) [22] and reliability plots [29]. Isotonic regression was used to recalibrate the probability from the XGBoost model in the calibration set to obtain more accurate predictions [30]. Furthermore, a decision curve analysis (DCA) was conducted to assess the potential benefit of guiding sepsis management based on predictions from our model across the threshold probabilities of 0 to 0.6. We set the upper limit of threshold probability at 0.6 because it is clinically unreasonable for a patient or doctor to accept a risk greater than 0.6 by balancing the harms of missing a patient with sepsis and unnecessary intervention on a patient without sepsis [31,32].

### Shapley Additive Explanation Algorithm

The Shapley additive explanation (SHAP) algorithm was used to show the average effect of each feature on the prediction model [33,34]. Bootstrapping was used to construct 95% CIs of the estimates using 1000 bootstrap samples of sepsis probabilities with replacement [23]. All computational analyses were conducted with Python (version 3.9.7; Python Software Foundation).

## Results

### Patient Characteristics

We obtained the medical records of 4603 trauma patients admitted to the ICU from MIMIC IV. After splitting the data randomly, there were 3222, 691, and 690 patients in the training, calibration, and testing sets, respectively. The 3 cohorts had similar characteristics, with a median age of 63 to 65 years and a higher proportion of males (ranging from 61% to 65%). The prevalence of sepsis in the above data sets was around 26% (Table 1).

**Table 1:** Characteristics of the trauma patients in the training, calibration, and testing sets.

Characteristics		All (N=4603)	Training set (n=3222)	Calibration set (n=691)	Testing set (n=690)
Age (years), median (IQR)		64 (42-81)	64 (42-81)	63 (44-81)	65 (42-82)
**Sex, n (%)**					
	Male	2878 (62.5)	2015 (62.5)	418 (60.5)	445 (64.5)
	Female	1725 (37.5)	1207 (37.5)	273 (39.5)	245 (35.5)
Charlson comorbidity index, median (IQR)		4 (1-5)	4 (1-5)	4 (1-5)	4 (1-5)
BMI (kg/m^2^), median (IQR)		26.9 (26.9-26.9)	26.9 (26.9-26.9)	26.9 (26.9-26.9)	27.9 (27.6-27.9)
Time interval from hospital to ICU^a^ admission (hours), median (IQR)		1 (0-1)	1 (0-1)	1 (0-1)	1 (0-2)
Length of stay in ICU (hours), median (IQR)		40 (21-82)	39 (21-82)	41 (21-84)	42 (21-81)
Sepsis, n (%)		1196 (26)	837 (26)	180 (26.1)	179 (25.9)

^a^ICU: intensive care unit.

### Model Evaluation and Model Calibration

In the test set, XGBoost outperformed logistic regression in both discrimination and calibration across all prediction windows (Table 2). For a prediction window of 6 hours, XGBoost had a higher AUROC (0.87, 95% CI 0.85-0.89), higher AUPRC (0.27, 95% CI 0.23-0.31) and lower ACE (0.33, 95% CI 0.31-0.35) than the logistic regression (AUROC=0.83, 95% CI 0.81-0.85; AUPRC=0.18, 95% CI 0.15-0.21; and ACE=0.44, 95% CI 0.44-0.45; Table 2, Multimedia Appendix 4). With longer prediction windows, the model discrimination as evaluated by AUROC or AUPRC decreased. The AUROC of the XGBoost model decreased from 0.88 (95% CI 0.86-0.90) in the 4-hour prediction window to 0.83 (95% CI 0.81-0.84) in the 24-hour window, and the AUROC of the logistic regression model decreased from 0.84 (95% CI 0.82-0.86) to 0.76 (95% CI 0.74-0.77). However, the model calibration improved slightly with an increase in the prediction window. The ACE of the XGBoost model decreased from 0.35 (95% CI 0.32-0.37) in the 4-hour prediction window to 0.30 (95% CI 0.28-0.32) in the 24-hour window, and the ACE of the logistic regression model decreased from 0.45 (95% CI 0.44-0.45) to 0.42 (95% CI 0.42-0.43; Table 2).

**Figure 2:**
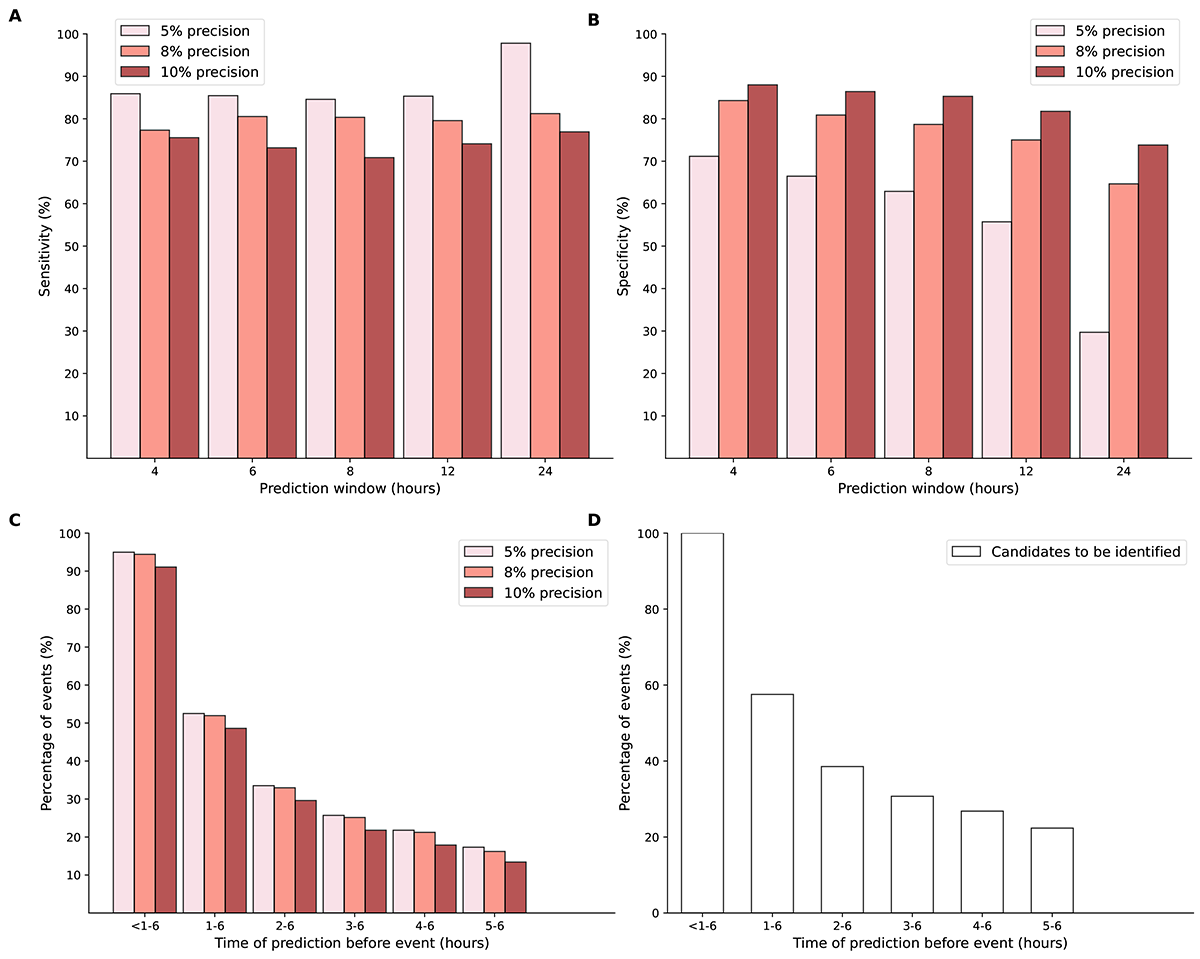
Time-step–level and outcome-level sensitivity and specificity by pairs of precision level (5%, 8%, and 10%) and prediction window for the Extreme Gradient Boosting (XGBoost) model. (A) Time-step–level sensitivity. (B) Time-step–level specificity. (C) Outcome-level sensitivity. (D) The proportion of (candidate) adverse events to be identified within each window.

**Figure 3:**
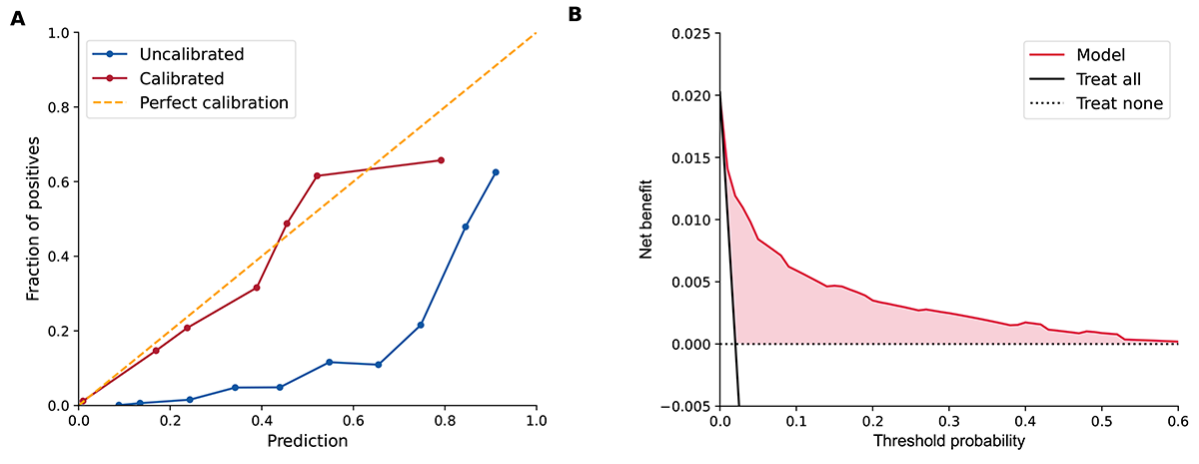
Calibration and clinical utility of the Extreme Gradient Boosting (XGBoost) model. (A) Calibration curves before and after calibration. (B) Decision curve.

At the time-step level, by using XGBoost, 73% (386/529) of sepsis-positive time steps were predicted at the 6-hour prediction window with a ratio of 9 false predictions for every true positive (10% precision), while 81% (428/529) of sepsis-positive time steps were predicted with a ratio of 12 false predictions for every true positive (8% precision; Figure 2). At the outcome level, the proportion of predicted sepsis episodes decreased with increased precision level. At the 10% precision level, XGBoost identified 91% (163/179) of sepsis events occurring in the subsequent 6 hours. Of note, the total number of events to be identified became fewer as the time period became shorter. There was a total of 22% (40/179) of patients for whom sepsis could be predicted 5 to 6 hours in advance, and XGBoost successfully predicted 60% (24/40) of them at the 10% precision level. The calibration curve showed that the predictions from XGBoost consistently overestimated the risk, whereas the predictions after recalibration lay snugly around the diagonal (Figure 3). The DCA demonstrated that XGBoost had a positive net benefit in clinical use for threshold probability across the threshold probabilities of 0 to 0.6 (Figure 3).

**Table 2:** Summary of model performance on the test set for Extreme Gradient Boosting (XGBoost) and logistic regression.

Performance metric		Value at 4 hours (95% CI)	Value at 6 hours (95% CI)	Value at 8 hours (95% CI)	Value at 12 hours (95% CI)	Value at 24 hours (95% CI)
**XGBoost**						
	AUROC^a^	0.88 (0.86-0.90)	0.87 (0.85-0.89)	0.86 (0.84-0.87)	0.84 (0.83-0.86)	0.83 (0.81-0.84)
	AUPRC^b^	0.27 (0.23-0.31)	0.27 (0.23-0.31)	0.26 (0.23-0.30)	0.25 (0.22-0.28)	0.23 (0.20-0.26)
	ACE^c^	0.35 (0.32-0.37)	0.33 (0.31-0.35)	0.32 (0.30-0.35)	0.32 (0.30-0.34)	0.30 (0.28-0.32)
**Logistic regression**						
	AUROC	0.84 (0.82-0.86)	0.83 (0.81-0.85)	0.81 (0.79-0.83)	0.79 (0.77 0.80)	0.76 (0.74-0.77)
	AUPRC	0.18 (0.15-0.22)	0.18 (0.15-0.21)	0.18 (0.15-0.21)	0.17 (0.14-0.20)	0.16 (0.14-0.18)
	ACE	0.45 (0.44-0.45)	0.44 (0.44-0.45)	0.44 (0.44-0.44)	0.44 (0.43-0.44)	0.42 (0.42-0.43)

^a^AUROC: area under the receiver operating characteristic curve.

^b^AUPRC: area under the precision-recall curve.

^c^ACE: average calibration error.

### SHAP Algorithm

**Figure 4:**
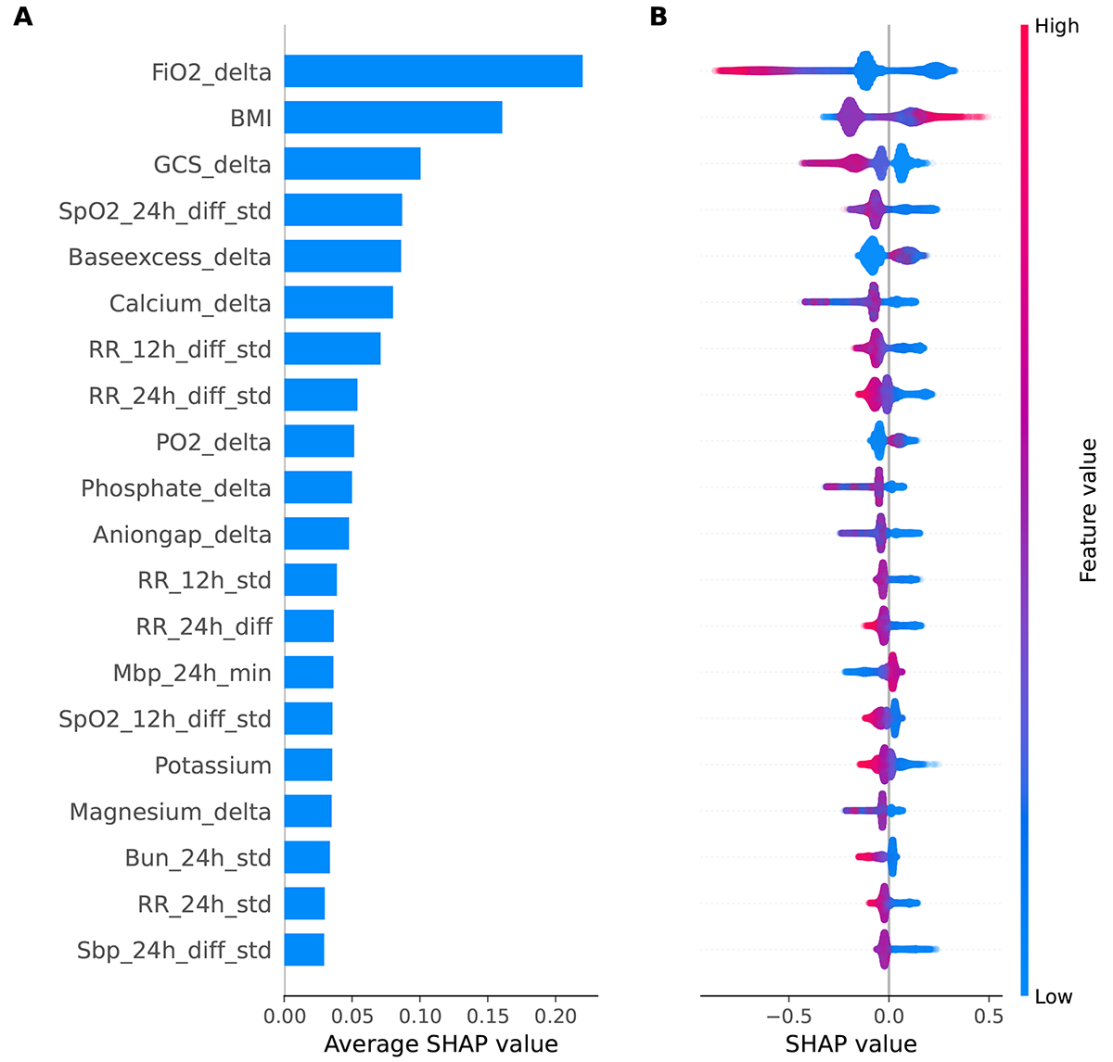
Bar plots showing (A) overall impacts of the top 20 features and (B) beeswarm plots showing impacts of the top 20 features across all patients. BMI: body mass index; Bun: blood urea nitrogen; delta: the latest measurement time gap; diff_std: differential standard deviation; diff: the difference between maximum and minimum values; FiO_2_: fraction of inspired oxygen; GCS: Glasgow Coma Scale; Mbp: mean blood pressure; PO_2_: arterial partial pressure of oxygen; RR: respiratory rate; Sbp: systolic blood pressure; SHAP: Shapley additive explanation; SpO_2_: saturation of peripheral oxygen; std: standard deviation.

When considering the relative importance of each feature in the model, we found that the latest measurement time gap of fraction of inspired oxygen (FiO_2_) had the greatest impact on the predictions, followed by BMI (Figure 4). Patients with a shorter measurement time gap of FiO_2_ or a higher BMI had an increased risk of sepsis. For time series variables of SD, differential SD, and the difference between maximum and minimum values of a feature, low values increased the risk of sepsis.

## Discussion

### Principal Findings

In this study, we developed an XGBoost risk prediction model to predict sepsis onset among trauma patients admitted to the ICU with a temporal resolution of 1 hour. This model achieved an AUROC ranging from 0.83 to 0.88 at the 4-to-24-hour prediction window. It predicted 73% (386/529) of sepsis-positive time-steps and 91% (163/179) of sepsis events in the subsequent 6 hours with a ratio of 9 false alerts for every true alert. Furthermore, the model achieved better discriminative and calibration performance than a traditional logistic regression model. However, this finding remains to be validated in other data sets; the classical logistic regression might be suboptimal compared with the XGBoost model.

Wong et al [10] recently reported that the widely applied ESM only identified sepsis before onset in 33% of patients, whereas our model identified up to 91% (163/179) of patients who developed sepsis in the subsequent 6 hours at 10% precision. To our knowledge, the XGBoost model in our study has better discrimination performance (with an AUROC of 0.87) than most previously published models that have been developed for real-time prediction of sepsis in the general ICU setting. Nemati et al [35] achieved an AUROC of 0.85 with a modified Weibull-Cox proportional hazards model for predicting sepsis 6 hours in advance, and Yang et al [28] achieved similar performance, also with the XGBoost algorithm. Kim et al [36] recently developed a type of deep learning model to predict sepsis that had higher discrimination performance than our model, with an AUROC of 0.91. However, their model could be seen as a complex black box due to its lack of interpretability, which might limit its acceptance among clinicians. Moreover, deep learning models like neural networks usually have a large number of parameters to estimate and have poor generalizability without sufficient training data, and it takes longer to train them than XGBoost [37]. The random forest model is another widely applied machine learning approach, but XGBoost might be a better option for imbalanced data sets, such as the one used in our study [38,39].

In addition to AUROC, a commonly reported measure of discriminative performance, we report AUPRC results for our model. This is more informative in class-imbalanced situations [22], such as sepsis prediction. Our model had an AUPRC of 0.27, which indicates low precision across a wide range of sensitivities in this extremely imbalanced data set. We found that the model achieved higher AUROC and AUPRC with shorter prediction windows. This could be attributed to the fact that a decreasing prediction window improved the timeliness of information, which boosted the predictive performance of the model. Moreover, we report calibration performance in addition to the commonly reported discriminative performance [40]. Calibration evaluates the agreement between the estimated and true risk of an outcome [41], which is important when a model is designed to make predictions at an individual level. Here, our model had an ACE of 0.33 before using isotonic regression calibration, which suggests that the model overestimated the risk of sepsis. However, model calibration decreased as the prediction window shortened, which might be associated with a decreasing number of positive steps due to the reduction of the prediction window. Several studies have reported a similar trend for AUROC across different prediction windows but have not reported changes in AUPRC or calibration [35,36]. Most importantly, a model with good discrimination and calibration performance does not necessarily have high clinical value [42]. Hence, DCA was used to assess the clinical utility of the model, and this showed a positive net benefit, suggesting that the model could help to inform timely treatment before sepsis onset in clinical practice. As the net benefit takes into account both true positives and false positives, the model with a net benefit is therefore worth choosing irrespective of the size or statistical significance of the benefit [42]. However, our model is not a practical tool at present, and we plan to develop a handy risk prediction tool by integrating the model into electronic health records for early identification of sepsis among trauma patients.

The matter of model applicability has not been well addressed in previous studies. In this study, we evaluated the time-step–level sensitivity and specificity of the model at different degrees of precision. The precision explicitly shows the number of false positives that the clinician encountered to identify one true positive episode or case. However, the sequential nature of making predictions determines the total number of positive steps; this does not directly correspond to the total number of patients with sepsis. Multiple positive time steps may be associated with a single sepsis episode. In fact, one positive prediction in the prediction window was enough to attract the attention of a clinician to make further decisions. Therefore, we calculated the outcome-level sensitivity (ie, the percentage of all sepsis episodes that had at least one correct prediction within a fixed time window before sepsis onset) to show the ability of the model to identify the percentage of true positive patients [43,44]. Furthermore, some previous studies have screened patients based on length of stay in the hospital, which might influence the generalizability and implementation of the model in a prospective setting [22,45].

Through SHAP analysis, we found that obese trauma patients were at an increased risk of sepsis. Obesity is associated with altered cellular immunity, increased use of central venous catheters because of difficulties with gaining peripheral access, and inadequate antibiotic dosing, all of which increase the risk of sepsis [46,47]. Moreover, obesity is associated with comorbidities like diabetes and hypertension, which have been identified as risk factors for sepsis [46,48]. Low values for time-series variables, such as differential SD of SpO_2_ and differential SD of respiratory rate, were associated with an increased risk of sepsis. One possible explanation was that most patients developed sepsis in a short time after admission. We compared the top 20 variables in the sorted SHAP value diagram in our model for critical trauma patients with those from other models developed for general critical patients and found that BMI ranked second for trauma patients but was not in the top 20 for general critical patients [28,49]. Contrarily, age ranked 14th for general critical patients but was not in the top 20 for trauma patients (Figure 4) [49].

### Limitations

This study has several limitations. First, though our model has shown good performance and clinical utility, it needs to be further validated at other medical centers. Second, the Injury Severity Score was not used for model development, even though this score is commonly used for assessing injury severity and might contain predictive information for sepsis in the trauma population. However, the Injury Severity Score is not available in the MIMIC database, and it is not an objective metric [50].

### Conclusions

In summary, an XGBoost model achieved high performance in both discrimination and calibration for continuous prediction of sepsis onset in the next 6 hours among trauma patients. Furthermore, the model was clinically useful and had a positive net benefit across the threshold probability.

## Appendix

Multimedia Appendix 1 List of raw variables (n=42) extracted from the MIMIC IV database. MIMIC IV: Medical Information Mart for Intensive Care IV.

Multimedia Appendix 2 The definition of the onset time of sepsis.

Multimedia Appendix 3 Details of the feature engineering.

Multimedia Appendix 4 Receiver operating characteristic (ROC) curves (A) and precision versus the sensitivity (PR) curves (B) for extreme gradient boosting (XGBoost) and logistic regression. AUROC: area under the receiver operating characteristic curve；AUPRC: area under the precision-recall curve.

## Abbreviations

ACE: average calibration error
AUPRC: area under the precision-recall curve
AUROC: area under the receiver operating characteristic curve
DCA: decision curve analysis
ESM: Epic Sepsis Model
FiO_2_: fraction of inspired oxygen
ICU: intensive care unit
MIMIC IV: Medical Information Mart for Intensive Care IV
SHAP: Shapley additive explanation
SOFA: Sequential Organ Failure Assessment
SpO_2_: saturation of peripheral oxygen
XGBoost: Extreme Gradient Boosting

## Data Availability

The Medical Information Mart for Intensive Care IV (MIMIC IV) data set used for analyses in this study has been published and made publicly available [18]. The code used in this study is largely based on the *numpy*, *pandas*, and *scikit-learn* libraries in python and is available from the corresponding authors upon reasonable request.
