# Supplementary material for: Real-Time Prediction of Sepsis in Critical Trauma Patients: Machine Learning–Based Modeling Study
Source: JMIR Form Res. 2023 Mar 31;7:e42452. doi: 10.2196/42452 (PMC10131736; doi:10.2196/42452)
Supplement: Multimedia Appendix 1 [file formative_v7i1e42452_app1.docx]

**List of raw variables (n = 42) extracted from the MIMIC IV database. MIMIC IV: Medical Information Mart for Intensive Care IV.**

| **Type** | **Variables** |
| --- | --- |
| Demographics and administrative features | Age, Sex, Charlson comorbidity index (CCI), Weight, Height, Hospital to ICU interval (Hospadmtime) |
| Vital signs | Heart rate (HR), Systolic blood pressure (Sbp), [Diastolic](javascript:;) [blood](javascript:;) [pressure](javascript:;) (Dbp), Mean blood pressure (Mbp), Respiratory rate, Saturation of peripheral oxygen (SpO_2_), Temperature, Glasgow Coma Scale (GCS) |
| Arterial blood gas | Saturation of arterial oxygen (SaO_2_), PO_2_, PCO_2_, PH, Base excess |
| Laboratory measurements | Hematocrit, Hemoglobin, Platelet count, White blood count (Wbc), Fraction of inspired oxygen (FiO_2_), International Normalized Ratio (INR), Prothrombin time (PT), Partial thromboplastin time (PTT), Neutrophils count, Chloride, Calcium, Potassium, Sodium, Albumin, Anion gap, Blood urea nitrogen (BUN), Creatinine, Magnesium, Phosphate, Bilirubin, Glucose, Lactate, End-tidal carbon dioxide (Etco_2_), [Alkaline](javascript:;) [phosphatase](javascript:;) |
