# Supplementary material for: Real-Time Prediction of Sepsis in Critical Trauma Patients: Machine Learning–Based Modeling Study
Source: JMIR Form Res. 2023 Mar 31;7:e42452. doi: 10.2196/42452 (PMC10131736; doi:10.2196/42452)
Supplement: Multimedia Appendix 2 [file formative_v7i1e42452_app2.docx]

**The definition of the onset time of sepsis:**

Sepsis was defined as the presence of both suspected infection and organ dysfunction [1, 2]. The time of suspected infection was defined as the earlier time of samples obtained (regardless of specific types) for culture purposes and antibiotic administration within a specific time. If the culture sampling was obtained first, the antibiotic had to be given within 72 h. If the antibiotic was given first, the culture sampling had to be obtained in the subsequent 24 h. The organ dysfunction was identified as an acute increase of two or more points in a Sequential Organ Failure Assessment (SOFA) score. The onset time of sepsis was then defined as the earlier time of suspected infection and acute increase of SOFA score≥2 in the prerequisite that the increase of SOFA score occurred within the timeframe of 48 h before to 24 h after suspected infection [3, 4].

**References**

1. Singer M, Deutschman CS, Seymour CW, Shankar-Hari M, Annane D, Bauer M, et al. The third international consensus definitions for sepsis and septic shock (sepsis-3). JAMA 2016 Feb 23;315(8):801-810. PMID: 26903338. doi: 10.1001/jama.2016.0287.

2. Seymour CW, Liu VX, Iwashyna TJ, Brunkhorst FM, Rea TD, Scherag A, et al. Assessment of clinical criteria for sepsis: For the third international consensus definitions for sepsis and septic shock (sepsis-3). JAMA 2016 Feb 23;315(8):762-774. PMID: 26903335. doi: 10.1001/jama.2016.0288.

3. Moor M, Horn M, Rieck B, Roqueiro D, Borgwardt K. Temporal convolutional networks and dynamic time warping can drastically improve the early prediction of sepsis. ArXiv Preprint ArXiv 2019 Feb 7;1902.01659.

4. Lauritsen SM, Thiesson B, Jorgensen MJ, Riis AH, Espelund US, Weile JB, et al. The framing of machine learning risk prediction models illustrated by evaluation of sepsis in general wards. NPJ Digit Med 2021 Nov 15;4(1):158. PMID: 34782696. doi: 10.1038/s41746-021-00529-x.
