# Supplementary material for: Real-Time Prediction of Sepsis in Critical Trauma Patients: Machine Learning–Based Modeling Study
Source: JMIR Form Res. 2023 Mar 31;7:e42452. doi: 10.2196/42452 (PMC10131736; doi:10.2196/42452)
Supplement: Multimedia Appendix 3 [file formative_v7i1e42452_app3.docx]

**Details of the feature engineering:**

| **Features** | **Description** |
| --- | --- |
| **Before missing value imputation** |  |
| Measurement pattern features (n = 37) | The latest measurement time gap for all raw variables except demographic variables |
| **After missing value imputation** |  |
| Scoring features (n = 7) | Total SOFA score and PaO_2_ / Fraction of inspired oxygen (FiO_2_), Glasgow Coma Scale (GCS) score, mean arterial pressure, serum creatinine, total bilirubin, platelets according to the SOFA scoring systems. |
| Time series variables (n = 441) | Maximum, minimum, mean, median, standard deviation (SD), differential SD, and difference of 21 variables [Glucose, Heart rate, Mean blood pressure (Mbp), Respiratory rate, Systolic blood pressure (Sbp), Saturation of peripheral oxygen (SpO_2_), Temperature, White blood count (Wbc), Creatinine, Platelet count, Saturation of arterial oxygen (SaO_2_), Partial thromboplastin time (PTT), Blood urea nitrogen (BUN), Calcium, Phosphate, Lactate, Hematocrit, Hemoglobin, Fraction of inspired oxygen (FiO_2_), [Alkaline](javascript:;) [phosphatase](javascript:;), total SOFA score] in prior 6/12/24 hours. |
