# Supplementary figures and images for: Real-Time Prediction of Sepsis in Critical Trauma Patients: Machine Learning–Based Modeling Study
Source: JMIR Form Res. 2023 Mar 31;7:e42452. doi: 10.2196/42452 (PMC10131736; doi:10.2196/42452)

**A**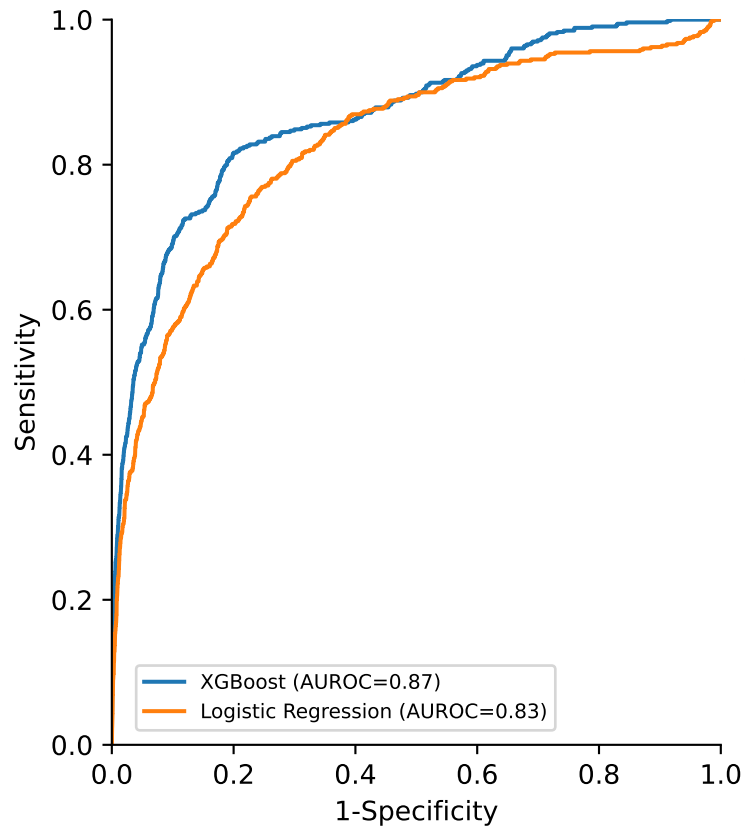**B**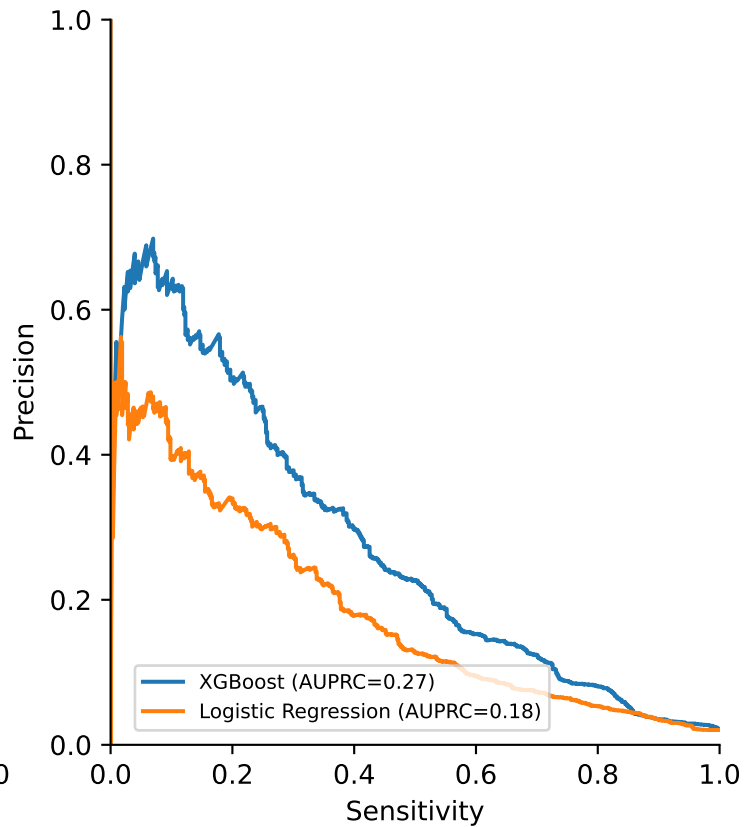

Supplement: Multimedia Appendix 4 [file formative_v7i1e42452_app4.pdf]
